# Birth of puppies after endoscopically guided transcervical intrauterine insemination with cryopreserved epididymal canine spermatozoa

**DOI:** 10.1186/s13028-025-00809-6

**Published:** 2025-05-22

**Authors:** Eva Axnér, Ulrika Hermansson

**Affiliations:** 1 Department of Clinical Sciences, Swedish University of Agricultural Sciences, P.O. Box 7054, Uppsala, SE 750 07 Sweden; 2 Small Animal Clinic, Swedish University of Agricultural Sciences, P.O. Box 7040, Uppsala, SE 750 07 Sweden

**Keywords:** Artificial insemination, Dog, Reproduction, Sperm

## Abstract

**Background:**

The preservation of epididymal spermatozoa is useful for saving important genetic material from valuable individuals who die suddenly or have to be castrated. The birth of puppies after artificial insemination with canine epididymal spermatozoa has been reported in only a few cases. Surgical insemination with frozen-thawed epididymal spermatozoa has resulted in pregnancies, but usually with low conception rates. Freshly collected and chilled epididymal canine semen has also resulted in conception after vaginal insemination. Considering the invasiveness of surgical insemination and the almost unlimited storage time of cryopreserved spermatozoa, transcervical intrauterine insemination with frozen-thawed epididymal spermatozoa would be beneficial. It has the potential to use genetic material that would otherwise be lost, both in domestic dogs and for the preservation of wild threatened canids.

**Case presentation:**

A 7-year-old, 20 kg male hunting dog was injured by a wild boar during hunting, and euthanasia was recommended for welfare reasons. Because the dog was a hunting champion in a numerically very small breed, the owner wanted to have spermatozoa preserved for future breeding. The dog was anaesthetised, both testes were removed, and the dog was thereafter euthanized. Spermatozoa from both caudae epididymides were released in a prewarmed Uppsala extender with the mincing method. The samples were routinely frozen with the Uppsala method. A half-filled straw was used for test thawing, resulting in 20% motile spermatozoa with slow progressive movement. Three years later, a 23-month-old bitch of the same breed was inseminated with endoscopically guided transcervical intrauterine sperm deposition. She was inseminated once, five days after a serum progesterone value of 6.9 nmol/mL was reached, and two days after a value of 24.8 nmol/mL was reached. The total amount of cryopreserved spermatozoa was used (a total dose of 1087 × 10^6^ spermatozoa and 217 × 10^6^ progressively motile spermatozoa remaining after test thawing). Eight puppies were born 59 days after insemination.

**Conclusions:**

Although rarely reported, artificial insemination with cryopreserved epididymal canine spermatozoa is an alternative in preserving valuable genetic animals when a male is injured beyond recovery.

## Background

The preservation of epididymal spermatozoa is useful for saving important genetic material from valuable individuals who die suddenly [1]. Domestic species, such as dogs and cats, are often used as models for the preservation of epididymal spermatozoa from wild species [2]. Dog owners sometimes want to preserve the breeding value of a male dog that has to be castrated or euthanized for welfare reasons. Although there are several reports on the collection and preservation of canine epididymal spermatozoa, the birth of puppies after artificial insemination with epididymal spermatozoa has been reported in only a few cases. The first birth of a puppy after surgical insemination with frozen-thawed epididymal spermatozoa was reported in a Boxer [3]. Spermatozoa were retrieved post-mortem from a male Boxer that was euthanized because of generalised seizures that did not respond to treatment. In another case, intravaginal insemination with fresh epididymal spermatozoa collected the day of insemination resulted in the birth of eight American Staffordshire Terrier puppies [4]. The male dog was castrated because of prostatic hyperplasia, and epididymal spermatozoa collected because an ejaculate collected by digital manipulation was haemorrhagic and oligozoospermic. Chilled epididymal spermatozoa collected post-mortem resulted in the birth of one Chihuahua puppy after vaginal insemination two days after sperm collection [5]. In a series of experiments involving surgical insemination of frozen-thawed epididymal canine spermatozoa, pregnancies were achieved in 13/60 (21.7%) of the bitches [6–8]. Epididymal spermatozoa differ from ejaculated spermatozoa in that they have not been exposed to seminal plasma, which is mainly composed of prostatic fluid (PF) in the dog. The contents of the PF may protect sperm cells, increasing their motility, survival, and fertilisation ability. When epididymal spermatozoa were exposed to PF, both post thaw motility and fertility increased compared with those of unexposed epididymal spermatozoa [6]. Cryopreservation has greater potential than the use of fresh or chilled epididymal spermatozoa to preserve the breeding value of a male dog, which for some reason cannot be used for mating anymore. However, frozen-thawed semen should be deposited in the uterus to obtain acceptable pregnancy rates. Transcervical insemination (TCI) is less invasive and is therefore preferred over surgical insemination, which is banned in several countries [9, 10]. TCI with frozen-thawed epididymal spermatozoa would therefore be beneficial both for domestic dogs and for the preservation of wild threatened canids but has not been previously described.

### Case presentation

A 7-year-old, 20 kg male hunting dog was injured by a wild boar during hunting in 2017. On arrival at the animal hospital, both hind legs were paralyzed. Radiography revealed herniated discs and bleeding over a large area in the back from T13 to L4. Because the dog was a hunting champion in a numerically very small breed, the owner was very eager to have spermatozoa preserved for future breeding. The collection of an ejaculate was not an alternative because of the dog’s condition. After anaesthesia with fentanyl and propofol intravenously, both testes were removed, and the dog was thereafter euthanized. Spermatozoa from both caudae epididymides were released in prewarmed Uppsala extender step 1 [11] by mincing the organs and thereafter removing the tissue. The sperm concentration was evaluated with a Bürker hemocytometer. A total of 1199 × 10^6^ spermatozoa were retrieved. Sperm morphology was evaluated by counting 100 spermatozoa fixed in formol-saline under a phase contrast microscope at 400x magnification, resulting in 13% morphologically defective spermatozoa. The samples were routinely frozen with the Uppsala method [11]. In brief, the extended sperm sample was cooled on a cooling bench, diluted with an equal volume of extender 2 to a final concentration of 457 × 10^6^ spermatozoa/mL, loaded in medium straws (0.5 mL) and frozen by stepwise lowering in a nitrogen tank. A half-filled straw was used for thawing. Subjective evaluation of motility revealed 20% motile spermatozoa with slow progressive movement. Three years after semen freezing, a 23-month-old primiparous bitch of the same breed was inseminated by endoscopically guided TCI (Karl Storz 43 cm endoscope, with 6° direction view). She was inseminated once, 5 days after a serum progesterone value of 6.9 nmol/mL was reached, and two days after a value of 24.8 nmol/mL was reached, evaluated with chemiluminescence (Immulite^®^ 2000, Siemens). Vaginal cytology was indicative of oestrus with mostly cornified epithelial cells. Because of the low progressive motility at test thawing and the previously reported low fertility of epididymal spermatozoa, the total amount of cryopreserved spermatozoa was used (a total dose of 1087 × 10^6^ spermatozoa, and 217 × 10^6^ progressively motile spermatozoa remaining after test thawing). Ultrasound evaluation 33 days later revealed several live embryos, and radiography on day 54 revealed eight well-mineralised foetuses. Eight puppies were born 59 days after insemination.

## Discussion and conclusions

Although rarely reported, artificial insemination with cryopreserved epididymal canine spermatozoa is an alternative for preserving genetic material when a valuable breeding male is injured beyond recovery. There is some controversy regarding the effect of PF on canine semen conservation. Positive effects of PF on cryopreserved canine epididymal spermatozoa was reported in one study [6]. In another study, sperm motility parameters were significantly increased after fresh canine epididymal spermatozoa were exposed to homologous PF [2]. However, after the epididymal spermatozoa frozen with the same method as those used in this study were thawed, there were no significant differences between PF-exposed and non-PF-exposed samples. Four hours after thawing, spermatozoa in the PF exposure group presented a significantly greater proportion of DNA damage, indicating a possible harmful effect of PF [2]. It is likely that collection of homologous PFs will usually not be possible in cases in which epididymal spermatozoa need to be preserved, as in this case. A possibility might be to use PF from another male, but hygienic aspects must then be considered. In addition, the beneficial effects of PF on cryopreserved epididymal canine spermatozoa are controversial. The motility of the frozen-thawed spermatozoa was very low compared with the expected results of ejaculated spermatozoa frozen with the same method and lower than that previously reported for epididymal spermatozoa [2, 12]. The sample was frozen with approximately double the sperm concentration compared with our routine protocol for ejaculated spermatozoa because of the low initial motility and to keep the insemination volume lower by using fewer straws. We used all of the cryopreserved semen to optimise the chance of success. Because of the acute clinical situation in this case, more sophisticated sperm analyses, such as CASA or fluorescence microscopy, were not performed. As sperm membrane integrity was not evaluated, some of the non-motile spermatozoa might have survived. The fact that the motility of epididymal spermatozoa can be enhanced by the addition of PF indicates that non-motile spermatozoa may be stimulated by exposure to PF and thus are alive [2]. The possibility that such spermatozoa may participate in fertilisation after exposure to the female genital tract cannot be excluded. This case confirms the usefulness of preserving epididymal spermatozoa from individuals for which future mating will not be an alternative. The non-invasive TCI was successful and resulted in the birth of eight puppies, thus potentially preserving the genetics of the male.

## Data Availability

The data used during the current study are based on medical records that cannot be shared without the consent of the owners. All relevant data are described in this case report. Further questions about the case will be answered by the corresponding author upon reasonable request and after the removal of data that may identify individual animals.

## Declarations

### Prior publication

The data included in this article have previously been published as abstracts in the Proceedings of the 25th International EVSSAR Congress - European Veterinary Society for Small Animal Reproduction, Barcelona, Spain, 27th --29th June 2024.
